# Advanced Imaging of the Right Heart and Pulmonary Circulation: The Role of Echocardiography and Cardiovascular Magnetic Resonance

**DOI:** 10.3390/jcm15207809

**Published:** 2026-10-10

**Authors:** Marta Kościuk, Karol Adam Kaminski, Remigiusz Kazimierczyk

**Affiliations:** 1 Department of Cardiology and Internal Diseases, Medical University of Bialystok, Curie-Sklodowskiej 24a, 15-276 Bialystok, Poland; martkos@interia.pl (M.K.); fizklin@wp.pl (K.A.K.); 2 Department of Population Medicine and Lifestyle Diseases Prevention, Medical University of Bialystok, Curie-Sklodowskiej 24a, 15-276 Bialystok, Poland

**Keywords:** right heart, echocardiography, cardiovascular magnetic resonance, pulmonary arterial hypertension

## Abstract

Echocardiography and cardiovascular magnetic resonance (CMR) have substantially advanced the non-invasive evaluation of the right heart and pulmonary circulation. This review provides a comprehensive overview of contemporary and emerging applications of these modalities across nine selected, clinically relevant right-heart and pulmonary vascular disorders including pulmonary arterial hypertension, pulmonary embolism, and more. We summarize established diagnostic criteria and prognostic parameters derived from both modalities, highlighting the complementary roles of echocardiography—as the first-line, real-time assessment tool—and CMR, which provides the reference standard for right ventricular volumetry, tissue characterization via late gadolinium enhancement (LGE) and parametric mapping, and quantitative flow assessment. Emerging techniques, including speckle-tracking and feature-tracking strain imaging, four-dimensional flow CMR, exercise CMR, and hybrid PET/MRI, are discussed with respect to their current evidence base and potential for integration into clinical practice. The potential of artificial intelligence-assisted image analysis to enhance diagnostic efficiency is also addressed. A multimodality imaging strategy, tailored to the clinical context, represents the current standard of care in right heart disease.

## 1. Introduction

Right ventricular (RV) dysfunction is increasingly recognized as an important determinant of symptoms, disease progression, and clinical outcomes across a broad spectrum of cardiovascular and pulmonary disorders. It may develop secondary to advanced left-sided heart disease or represent a primary manifestation of conditions directly affecting the right heart or pulmonary circulation. Despite its established diagnostic and prognostic relevance, accurate assessment of RV structure and function remains one of the major challenges in contemporary cardiovascular imaging [1].

In contrast to the left ventricle (LV), the RV is characterized by complex three-dimensional geometry, a heavily trabeculated endocardial surface, and a multidirectional contraction pattern, which limit its assessment by conventional two-dimensional (2D) echocardiography. Routinely used indices of RV systolic function, including tricuspid annular plane systolic excursion (TAPSE), peak systolic velocity of the tricuspid annulus (RV s′), and fractional area change (FAC), reflect only selected components of RV mechanics and may therefore fail to detect subtle or early myocardial dysfunction. Moreover, RV performance is highly dependent on loading conditions, particularly afterload, further complicating the interpretation of conventional functional parameters in conditions associated with pressure or volume overload [2].

Advances in cardiovascular imaging have enabled a more comprehensive characterization of RV morphology, function, and myocardial mechanics [3]. Speckle-tracking echocardiography provides quantitative assessment of myocardial deformation, with RV longitudinal strain emerging as a sensitive marker of subclinical dysfunction and a potentially valuable tool for risk stratification [2]. Cardiac magnetic resonance (CMR), regarded as the reference standard for the quantitative assessment of RV volumes and ejection fraction (RVEF), additionally provides myocardial tissue characterization through techniques such as late gadolinium enhancement (LGE), T1 mapping, and extracellular volume (ECV) quantification [4]. These approaches may provide complementary diagnostic and prognostic information beyond conventional measures of RV systolic function.

This review provides a clinically focused overview of contemporary and emerging applications of echocardiography and CMR in the assessment of the right heart and pulmonary circulation. By integrating echocardiographic and CMR findings within a common clinical framework, we highlight not only established diagnostic and prognostic parameters but also emerging techniques, including myocardial deformation imaging, parametric mapping, 4D-flow and exercise CMR, hybrid PET/MRI, and AI-assisted image analysis. Emphasis is placed on recent evidence regarding their potential role in early detection of RV dysfunction, phenotyping, risk stratification, and monitoring of therapeutic response. Thus, this review is intended to complement, rather than replace, comprehensive guidelines by providing practicing clinicians with a focused overview of contemporary and emerging right-heart imaging tools.

This narrative review was informed by a structured literature search of PubMed/MEDLINE covering publications till August 2026.

## 2. Pulmonary Arterial Hypertension

Pulmonary arterial hypertension (PAH) is a progressive pulmonary vasculopathy defined by a mean pulmonary arterial pressure > 20 mmHg with a pulmonary artery wedge pressure ≤ 15 mmHg and pulmonary vascular resistance (PVR) > 2 Wood Units, leading to progressive RV pressure overload, maladaptive remodeling, and ultimately right heart failure. Right heart catheterization (RHC) remains the gold standard for the diagnosis and hemodynamic classification of PH. Echocardiography represents the principal first-line non-invasive modality for estimating the probability of PAH and characterizing its cardiac consequences, whereas CMR provides complementary information on RV morphology, function, flow, and myocardial tissue characteristics but does not establish the hemodynamic diagnosis of PAH [5]. Advanced cardiovascular imaging has transformed the evaluation of PAH by enabling comprehensive non-invasive characterization of right heart morphology, function, and myocardial remodeling with direct diagnostic, prognostic, and therapeutic implications.

Echocardiographic assessment of PAH requires a comprehensive multiparametric approach integrating right heart morphology, RV systolic function, and indirect hemodynamic indices. Structural signs suggestive of RV pressure overload include RV enlargement, a basal RV-to-LV diameter ratio > 1.0, and interventricular septal flattening resulting in a characteristic D-shaped left ventricle. Right atrial (RA) enlargement, defined by an end-systolic RA area > 18 cm^2^, as well as inferior vena cava (IVC) dilatation with reduced inspiratory collapse, may indicate elevated right-sided filling pressures. RV systolic dysfunction can be identified by a reduced FAC < 35%, decreased TAPSE < 17 mm, and reduced S′ < 9.5 cm/s. Assessment of the pulmonary circulation includes measurement of peak tricuspid regurgitation velocity (TRV), with values > 2.8 m/s suggesting an increased probability of PH, although TRV should not be interpreted in isolation. Moreover, TRV may underestimate pressure gradients, particularly in severe tricuspid regurgitation, or overestimate them in high-output states and due to technical or measurement errors. Additional Doppler findings include shortening of the RV outflow tract acceleration time (RVOT AT < 105 ms) and the presence of mid-systolic notching, which may support the presence of pre-capillary PH. Systolic pulmonary artery pressure (sPAP) can be estimated from the tricuspid regurgitation pressure gradient combined with estimated RA pressure. Finally, pericardial effusion may be observed in advanced PH and represents an important marker of disease severity [2,5].

Beyond conventional echocardiographic indices, advanced imaging techniques provide a more comprehensive assessment of RV function and its interaction with the pulmonary circulation. RV free-wall longitudinal strain (RVFWLS), derived from speckle-tracking echocardiography, enables quantitative assessment of myocardial deformation and may detect subtle RV systolic dysfunction before abnormalities become apparent using conventional parameters [2]. Three-dimensional (3D) echocardiography allows volumetric assessment of the RV without relying on geometric assumptions and enables estimation of RV volumes and RVEF, thereby overcoming some of the limitations imposed by the complex RV geometry [2,6]. RVEF and RV volumes derived from real-time three-dimensional (RT3DE) echocardiography show good agreement with CMR measurements. Among echocardiographic indices of RV systolic function, RV global longitudinal strain (RVGLS) demonstrates a strong correlation with CMR-derived RVEF and may facilitate the detection of RV dysfunction [6]. In addition, the TAPSE/sPAP ratio has emerged as a simple non-invasive surrogate of RV–pulmonary arterial (RV–PA) coupling, reflecting the relationship between RV contractile function and pulmonary arterial afterload [2]. These advanced parameters complement conventional echocardiographic measures and may provide additional diagnostic and prognostic information in patients with pulmonary hypertension (PH) [2,7]. Contemporary imaging increasingly shifts from isolated assessment of RV systolic function toward evaluation of RV–PA coupling [2]. It must be underlined that RV–PA coupling can be assessed at different levels of physiological and methodological complexity. The reference construct is derived from invasive pressure–volume analysis and is commonly expressed as the ratio of RV end-systolic elastance to arterial elastance (Ees/Ea), reflecting ventricular contractility relative to afterload. Non-invasive indices such as TAPSE/sPAP, RVGLS/sPAP, and RVEF/sPAP should be always considered surrogate markers of RV–PA coupling rather than direct measurements.

In current clinical practice, the principal strength of CMR in PAH lies in accurate and reproducible assessment of RV volumes, indexed RV end-diastolic and end-systolic volumes, RVEF, stroke volume and stroke-volume index, together with right atrial size and function. Serial CMR assessment enables quantification of RV reverse remodeling and changes in ventricular performance over time, and these conventional volumetric and functional parameters have substantially stronger clinical and prognostic validation than newer tissue-characterization biomarkers. Advanced techniques such as feature-tracking strain, LGE, T1 mapping, and ECV should therefore be regarded as complementary and potentially incremental rather than replacements for established CMR measures.

Beyond conventional volumetric assessment, CMR feature-tracking enables quantitative evaluation of RV myocardial deformation. CMR-derived RVGLS may provide additional information on myocardial contractile function and can be integrated with measures of pulmonary vascular load to assess RV–PA interaction. In patients with PAH, indices combining CMR-derived RVGLS with RHC-derived sPAP, pulmonary vascular resistance, or pulmonary arterial compliance have been associated with RV function and clinical outcomes. In particular, the hybrid RVGLS/sPAP ratio has shown promising prognostic value, suggesting that assessment of RV contractile performance relative to pulmonary vascular afterload may provide incremental information beyond isolated measures of RV function. These findings support a more comprehensive approach to CMR assessment of the right heart that integrates myocardial deformation with the hemodynamic burden imposed by the pulmonary circulation [8].

CMR parametric mapping extends conventional morphological and functional assessment by enabling quantitative characterization of myocardial tissue composition. Native T1 mapping is sensitive to alterations in both intracellular and extracellular myocardial compartments, whereas ECV fraction provides a quantitative estimate of extracellular matrix expansion and may serve as a surrogate marker of diffuse myocardial fibrosis [4].

Native T1 values have been shown to be significantly increased at the interventricular insertion regions in patients with precapillary PH, whereas no comparable regional differences are observed in healthy subjects. Increased native T1 at these regions was associated with greater RV end-diastolic and end-systolic volumes, lower RVEF, higher right atrial pressure, and increased NT-proBNP levels, suggesting a relationship between tissue alterations and the severity of RV remodeling. These changes may reflect localized fibrosis and/or oedema resulting from increased mechanical stress at the RV insertion points (RVIPs). Importantly, similar T1 abnormalities have been observed across different forms of precapillary PH, suggesting that they may reflect the consequences of chronic RV pressure overload rather than disease-specific myocardial involvement [9].

T2 mapping primarily reflects changes in myocardial water content and may therefore identify myocardial oedema and inflammation. These techniques may provide complementary information on myocardial remodeling beyond conventional measurements of RV volumes and RVEF. However, direct parametric assessment of the RV myocardium remains technically challenging because of the thin RV free wall and associated partial-volume effects, highlighting the need for high-resolution mapping techniques [4].

ECV mapping provides quantitative assessment of extracellular matrix expansion and may serve as a non-invasive marker of diffuse myocardial remodeling in PH [10]. ECV abnormalities are particularly prominent at the RVIPs, including the anterior and inferior septal insertion regions, and within the RV free wall [10,11]. Increased RV ECV has also been demonstrated in the interventricular septum, whereas comparable changes are less evident in the LV free wall [12]. In patients with PH, increased RV ECV is associated with impaired RV systolic performance, including lower RVEF and abnormalities in echocardiographic indices of RV function [11]. Experimental studies further suggest a spatial and temporal pattern of remodeling, with ECV expansion appearing relatively early at the RVIPs and subsequently progressing within the RV free wall and interventricular septum. Importantly, RV free-wall ECV correlates closely with histologically quantified collagen deposition, supporting its potential role as an imaging marker of diffuse interstitial fibrosis and adverse RV remodeling [12].

LGE is a common CMR finding in PH and typically localizes to the RVIPs, with more extensive enhancement occasionally extending into the interventricular septum. This characteristic distribution is thought to reflect focal myocardial remodeling related to chronic RV pressure overload and increased mechanical stress at the ventricular insertion regions [13]. The presence and extent of LGE are associated with adverse RV remodeling and hemodynamic impairment [13,14]. In PAH, quantitative assessment of LGE burden at the RVIPs, expressed as the LGE mass index, has been associated with higher mean pulmonary artery pressure and pulmonary vascular resistance, as well as lower RVEF and impaired RV longitudinal deformation. Greater LGE burden has also been observed in patients experiencing clinical deterioration or death, suggesting potential prognostic value [14]. However, the prognostic significance of LGE remains uncertain, as qualitative assessment of its presence or septal extension has not consistently provided independent prognostic information beyond established markers of RV dysfunction [13]. Thus, quantitative assessment of LGE extent may be more informative than the simple presence or absence of enhancement [13,14].

## 3. Pulmonary Embolism

In acute pulmonary embolism (PE), computed tomography pulmonary angiography (CTPA) and transthoracic echocardiography (TTE) represent the principal imaging modalities. CTPA remains the cornerstone for confirming the diagnosis and defining thromboembolic burden, whereas echocardiography provides rapid bedside assessment of the hemodynamic consequences of acute pulmonary vascular obstruction, particularly RV dilatation and dysfunction. Acute elevation of RV afterload may result in RV dilatation, increased wall stress, impaired systolic function, interventricular septal displacement, and, in severe cases, reduced LV filling and cardiac output [15]. In addition to conventional indices of RV dysfunction, several characteristic echocardiographic findings may support PE as the underlying etiology. The McConnell sign, defined as akinesis or marked hypokinesis of the RV free wall with preserved apical contractility, is considered a relatively specific finding suggestive of acute PE [15,16]. Another characteristic pattern is the “60/60 sign”, defined by a pulmonary artery acceleration time < 60 ms in combination with an estimated sPAP < 60 mmHg [15]. Although these signs may strengthen the suspicion of acute PE in the appropriate clinical context, contemporary echocardiographic assessment increasingly moves beyond qualitative findings toward quantitative characterization of RV dysfunction and its prognostic consequences [16,17].

RVGLS and RVFWLS may identify impaired RV contractility that is not fully captured by conventional indices such as TAPSE or FAC [16,18]. In patients with intermediate-risk PE, impaired RVGLS has been associated with increased short-term mortality and has shown incremental prognostic value over conventional echocardiographic and CT-derived parameters [18]. Importantly, combining RVGLS with conventional measures of RV enlargement, particularly the RV/LV diameter ratio, may further improve risk stratification, supporting a multiparametric approach rather than reliance on a single marker of RV dysfunction [19].

3D echocardiography provides additional advantages by allowing volumetric assessment of the RV without relying on geometric assumptions [20,21]. In acute intermediate-risk PE, 3D-derived RVEF and RV longitudinal strain are reduced compared with healthy controls and have been associated with adverse clinical outcomes. Longitudinal observations have also demonstrated different patterns of recovery among RV parameters, with myocardial deformation improving earlier in some patients, whereas abnormalities in 3D RVEF may persist for several months [20]. These findings suggest that advanced echocardiographic techniques may be useful not only for initial risk assessment but also for characterization of RV reverse remodeling following acute PE [20,21].

Assessment of the RA represents another emerging area of interest. Acute elevation of RV filling pressure and impaired RV systolic function directly affect RA mechanics. Speckle-tracking studies have demonstrated impaired RA deformation in acute PE, indicating that hemodynamic consequences extend beyond the RV itself [22]. More recent four-chamber strain analyses have shown progressive impairment of RVFWLS as well as RA reservoir and conduit strain with increasing PE severity [23]. Moreover, RA strain parameters provided prognostic information beyond conventional risk assessment, suggesting that evaluation of the right heart as an integrated RA–RV unit may offer additional insights into the severity of acute pressure overload [22,23].

CMR remains the reference technique for quantitative assessment of RV volumes and systolic function but currently has a limited role in the acute clinical evaluation of PE because of longer acquisition times, availability, and the clinical condition of affected patients [15,24]. Nevertheless, CMR represents a promising research tool for detailed assessment of RV remodeling and pulmonary flow. Prospective data have demonstrated the feasibility of CMR in patients with intermediate-risk PE and its ability to detect measurable changes in right-heart function and pulmonary artery flow before and after catheter-directed therapy [24]. Accordingly, CMR may be particularly valuable for investigating treatment response, persistent RV dysfunction, and mechanisms of post-PE remodeling rather than for routine acute diagnosis [24,25].

Beyond functional assessment, emerging CMR tissue-characterization techniques may provide insights into myocardial remodeling following PE [25,26]. In patients with persistent dyspnea after PE but without CTEPH, higher native T1 and ECV have been demonstrated within the interventricular septum compared with asymptomatic PE survivors, suggesting persistent interstitial myocardial remodeling [25]. More pronounced tissue abnormalities are observed in CTEPH, where elevated native T1 and ECV occur particularly at the RVIPs and interventricular septum, sometimes even in the absence of LGE, and correlate with RV dysfunction and pulmonary hemodynamic impairment. Importantly, native T1 values decrease following balloon pulmonary angioplasty, supporting a potential role for parametric mapping in monitoring reverse myocardial remodeling [26].

## 4. Chronic Thromboembolic Pulmonary Hypertension

Chronic thromboembolic pulmonary hypertension (CTEPH) is a distinct and potentially curable form of PH (WHO Group 4) that develops from incomplete resolution of acute pulmonary emboli, resulting in organized fibrous obstruction of the pulmonary vasculature and progressive RV pressure overload. The estimated incidence of CTEPH ranges from 0.5% to 2% following an acute PE episode, and CTEPH represents one of the few etiologies of PH amenable to surgical cure by pulmonary endarterectomy (PEA).

The diagnostic evaluation of suspected CTEPH requires integration of perfusion imaging, cross-sectional vascular imaging, and invasive hemodynamic assessment. Ventilation/perfusion (V/Q) scintigraphy remains the most effective screening modality for excluding chronic thromboembolic pulmonary disease; in the absence of significant parenchymal lung disease, a normal perfusion scan has a negative predictive value of approximately 98% for excluding CTEPH [5]. CTPA is subsequently used to identify and anatomically characterize chronic thromboembolic lesions, including webs, bands, eccentric wall-adherent thrombi, vascular narrowing or occlusion, and mosaic perfusion. Importantly, a negative CTPA does not completely exclude distal chronic thromboembolic disease. RHC remains essential for hemodynamic confirmation and classification of PH, whereas digital subtraction angiography may provide additional anatomical information when CTPA is inconclusive and is particularly relevant for assessment of treatment options and procedural planning for BPA [5].

Advanced cardiovascular imaging may also play an important role throughout the clinical pathway—from initial hemodynamic characterization and risk stratification to post-interventional monitoring of RV reverse remodeling [2].

TTE is the recommended initial screening modality, providing indirect evidence of PH through elevated peak tricuspid regurgitant jet velocity (TRV ≥ 2.8 m/s), RV dilatation, septal flattening (D-sign, eccentricity index), and reduced TAPSE. The RVOT AT—and the mid-systolic notching pattern reflecting pulsatile impedance from organized proximal thromboembolic obstruction—may provide additional discriminatory value over other forms of precapillary PH [2,5]. Speckle-tracking echocardiography has demonstrated prognostic relevance in CTEPH [27], with RVGLS of free wall independently predicting clinical outcomes both before and after PEA. Exercise stress echocardiography may further unmask residual RV–PA uncoupling in patients with persistent exertional limitation despite technically successful intervention [28].

CMR provides accurate and highly reproducible non-invasive assessment of RV volumes, mass, and systolic function in CTEPH and is particularly valuable for quantifying RV remodeling and its evolution following treatment [5]. Serial CMR can document reverse remodeling through changes in RVEDVI, stroke volume, and RVEF, while phase-contrast imaging provides quantitative assessment of pulmonary arterial flow and derived flow-related parameters. However, CMR does not replace invasive hemodynamic assessment, as no established CMR method currently allows reliable estimation of pulmonary artery pressure; RHC therefore remains the reference standard for hemodynamic characterization [5,29]. Furthermore, phase-contrast flow CMR enables non-invasive quantification of net pulmonary blood flow, pulmonary arterial compliance, and right heart output [5]. LGE at the RVIPs, reflecting focal pressure-overload fibrosis, is a recognized CMR finding in advanced CTEPH and may persist after technically successful PEA, with potential implications for long-term RV functional recovery [26].

## 5. Heart Failure with Preserved Ejection Fraction

Heart failure with preserved ejection fraction (HFpEF) represents a heterogeneous clinical syndrome characterized by symptoms and signs of heart failure in the presence of a left ventricular ejection fraction (LVEF ≥ 50%), diastolic dysfunction, and elevated left-sided filling pressures [30]. Although historically considered a disease predominantly of the left ventricle, emerging evidence underscores the central role of RV dysfunction and impaired RV-PA coupling in determining functional capacity, symptom burden, and adverse clinical outcomes in this population [31,32].

RV involvement is increasingly recognized as an important component of the HFpEF phenotype, particularly in patients who develop PH [32]. 2D speckle-tracking echocardiography enables quantitative assessment of RV myocardial deformation and may identify clinically relevant systolic dysfunction that is not adequately captured by conventional parameters. In patients with HFpEF, RVGLS is significantly impaired compared with healthy controls and shows a good correlation with CMR-derived RVEF. Importantly, impaired RVGLS provides incremental prognostic information for mortality and heart-failure hospitalization, whereas abnormal TAPSE or FAC alone may have more limited prognostic value [33].

These findings also support a multiparametric rather than single-parameter approach to RV assessment. In the PARAGON-HF echocardiographic sub study, RV dysfunction defined using TAPSE, FAC, and RVFWLS was common, affecting more than half of patients with HFpEF. Importantly, the presence of at least two abnormal RV parameters was associated with a substantially higher risk of heart-failure hospitalization and cardiovascular death, whereas an isolated abnormal parameter was not independently associated with adverse outcomes. Thus, integration of conventional measures with myocardial deformation imaging may better characterize the heterogeneous spectrum of RV dysfunction in HFpEF [31].

An important advance in HFpEF imaging is the assessment of RV–PA coupling. The TAPSE/sPAP ratio provides a simple non-invasive estimate/surrogate of RV contractile performance relative to pulmonary afterload. In HFpEF, lower TAPSE/sPAP is associated with higher pulmonary vascular resistance, reduced pulmonary arterial compliance, impaired exercise capacity, and worse outcomes [32]. Its prognostic relevance has also been confirmed in meta-analyses, although reported cut-off values vary between studies. Thus, RV–PA coupling may provide clinically relevant information beyond isolated assessment of RV systolic function [34].

Exercise echocardiography may further uncover abnormalities of the right heart that are not apparent at rest. Patients with HFpEF may exhibit relatively preserved resting RV systolic mechanics but develop an inadequate increase in RV contractility during exercise, while simultaneously experiencing a disproportionate rise in pulmonary pressures and reduction in pulmonary arterial compliance [35]. Invasive exercise studies have confirmed that RV–PA uncoupling during exertion reflects both impaired RV contractile reserve and abnormal pulmonary vascular responses, particularly in patients with exercise-induced pulmonary vascular dysfunction [36]. Thus, dynamic assessment of RV–PA interaction may be particularly relevant in HFpEF, in which resting imaging can underestimate the severity of right-heart dysfunction [35,36].

3D echocardiography represents a further step toward a more comprehensive assessment of RV performance by enabling direct volumetric quantification of RV volumes and RVEF without relying on the geometric assumptions inherent to two-dimensional imaging [2]. More recently, 3D-derived RVEF has also been incorporated into assessment of RV–PA coupling. In patients with HFpEF, the 3D RVEF/sPAP ratio was independently associated with cardiovascular death or heart-failure hospitalization and demonstrated greater prognostic discrimination than the conventional TAPSE/sPAP ratio. These findings suggest that volumetric assessment of RV contractile function relative to pulmonary afterload may provide a more comprehensive characterization of RV–PA coupling than indices based solely on longitudinal annular motion [37].

Overall, contemporary echocardiographic assessment of the right heart in HFpEF is evolving from isolated measurements of RV systolic function toward an integrated evaluation incorporating myocardial deformation, multidimensional RV function, RV–PA coupling, and dynamic exercise reserve. Such an approach may improve identification of clinically relevant RV dysfunction, refine HFpEF phenotyping, and enhance prognostic stratification [31,32].

The prognostic relevance of CMR-derived RVEF has been demonstrated across the spectrum of heart failure [38]. More recently, CMR feature-tracking has extended RV assessment beyond conventional volumetric measures by enabling quantitative characterization of myocardial deformation. In a large HFpEF cohort, RVGLS and global circumferential strain (RVGCS) were independently associated with all-cause mortality and improved prognostic discrimination when added to conventional clinical and CMR parameters. Importantly, their prognostic value persisted after adjustment for RVEF and, in patients with available parametric mapping, after consideration of native T1 and ECV, supporting the incremental role of RV deformation imaging in HFpEF [39].

CMR also enables detailed assessment of RA function and RA–RV interaction, providing information that may not be apparent from RVEF alone. In compensated HFpEF, impaired early RV filling has been demonstrated despite preserved RV systolic function and was accompanied by reduced RA conduit function and a compensatory increase in RA booster-pump function. Impaired early RV filling was closely related to reduced exercise capacity, suggesting that abnormalities of RV diastolic filling and RA–RV interaction may precede overt RV systolic dysfunction [40]. CMR feature-tracking further allows quantification of phasic RA deformation. In HFpEF patients in sinus rhythm, impaired RA reservoir and conduit strain were independently associated with heart-failure hospitalization and cardiovascular death, even after adjustment for established risk markers, including RVEF. In contrast, these parameters lost their prognostic value in patients with persistent atrial fibrillation, emphasizing the influence of rhythm on the interpretation of RA mechanics [41].

Beyond functional assessment, parametric CMR may provide insights into RV tissue remodeling. High-resolution T1 mapping enables assessment of the thin RV free wall and calculation of RV ECV as a marker of extracellular matrix expansion. In patients with PH associated with HFpEF (PH-HFpEF), increased RV ECV was associated with greater RV end-diastolic volume and impaired RV free-wall strain. Notably, RV ECV was comparable to that observed in PAH despite a lower pulmonary vascular load and, unlike in PAH, was not related to RV afterload. These findings suggest that diffuse RV interstitial remodeling in PH-HFpEF may develop partly independently of pulmonary vascular load and may reflect additional myocardial or systemic mechanisms contributing to RV dysfunction. However, given the small sample size and technical challenges associated with RV free-wall T1 mapping, these findings require further validation [42].

## 6. Arrhythmogenic Right Ventricular Cardiomyopathy

Arrhythmogenic right ventricular cardiomyopathy (ARVC) is an inherited myocardial disorder characterized by progressive replacement of ventricular myocardium by fibrofatty tissue, predisposing to ventricular arrhythmias, sudden cardiac death, and, in advanced stages, RV failure [43]. Multimodality cardiovascular imaging plays a central role in the diagnosis and risk stratification of ARVC according to the revised Task Force Criteria, providing complementary information on structural remodeling, myocardial deformation, tissue characterization, and disease activity [43,44].

Advanced echocardiography provides a more sensitive and quantitative characterization of right-heart involvement in ARVC. 3D echocardiography enables direct assessment of RV volumes and RVEF, demonstrating progressive RV dilatation and reduction in RVEF with increasing disease severity. Importantly, 3D echocardiography improves detection of regional wall-motion abnormalities compared with conventional 2D echocardiography, although 3D echocardiography tends to underestimate RV volumes relative to CMR [45]. Speckle-tracking echocardiography provides additional information on regional and global RV myocardial mechanics. RV longitudinal strain becomes less negative with disease progression, reflecting impaired myocardial shortening, while regional deformation abnormalities may be detectable before overt RV dilatation or global systolic dysfunction [46,47]. In asymptomatic pathogenic mutation carriers, reduced peak systolic strain and post-systolic shortening have been demonstrated predominantly in the basal and mid RV free wall, particularly in the sub tricuspid region [46]. In more advanced disease, worsening RV deformation is accompanied by increased mechanical dispersion, reflecting greater temporal heterogeneity of RV contraction. Both abnormal RV deformation patterns and increased mechanical dispersion have been independently associated with life-threatening ventricular arrhythmias, suggesting that deformation imaging may contribute not only to early disease detection but also to arrhythmic risk stratification [47]. RA mechanics are also impaired in ARVC. RA reservoir, conduit, and booster-pump strain are reduced compared with healthy individuals, and these abnormalities may be present even when RA volume remains normal, suggesting that atrial deformation may identify early RA involvement. Furthermore, both RVGLS and RAGLS become more impaired in patients who subsequently experience sustained ventricular arrhythmias or cardiovascular death [48].

CMR plays a central role in ARVC by combining accurate assessment of RV volumes, RVEF, and regional wall motion with myocardial tissue characterization [44]. CMR feature-tracking provides quantitative assessment of myocardial deformation, and impaired RVGLS has demonstrated both diagnostic and prognostic value, including an independent association with subsequent adverse cardiovascular events [49]. LGE enables visualization of focal myocardial fibrosis and has gained an important role in contemporary diagnostic criteria for arrhythmogenic cardiomyopathy. In the Padua criteria, transmural LGE with a stria-like pattern involving at least one RV region represents a major structural criterion when confirmed in two orthogonal planes. However, the sensitivity of RV LGE remains limited by the thin RV free wall and spatial-resolution constraints; therefore, absence of RV LGE does not exclude the disease. Importantly, the presence of LGE has also been associated with ventricular arrhythmias and adverse outcomes, supporting its potential prognostic relevance beyond morphological assessment alone [44].

Parametric mapping may further extend CMR assessment beyond focal fibrosis detected by LGE. Native T1 mapping is sensitive to diffuse myocardial abnormalities and has shown increased values in patients with overt ARVC; however, direct assessment of the RV free wall remains technically challenging because of its limited thickness and partial-volume effects, and several studies have therefore focused primarily on the left ventricle or septum [50]. ECV provides complementary information on diffuse extracellular matrix expansion. Recent data indicate that increased myocardial ECV, together with impaired RV and LV GLS, is independently associated with sustained ventricular arrhythmias and adds prognostic value beyond established ARVC risk scores [51]. Thus, the combination of feature-tracking and parametric mapping may identify diffuse myocardial involvement not fully captured by conventional cine imaging or focal LGE assessment [50,51].

T2-based imaging may be particularly relevant during the inflammatory “hot phase” of arrhythmogenic cardiomyopathy, characterized by chest pain, myocardial injury, and a myocarditis-like clinical presentation. During such episodes, CMR may demonstrate myocardial oedema or hyperemia together with LGE, reflecting active myocardial injury superimposed on chronic fibrotic remodeling. However, these abnormalities frequently involve the left ventricle and are particularly associated with specific arrhythmogenic cardiomyopathy phenotypes rather than isolated classical right-dominant ARVC [52]. Consequently, T2 mapping should currently be regarded as an emerging tool for identifying active inflammatory injury rather than a routine diagnostic marker of chronic ARVC [44].

Overall, contemporary imaging of ARVC is evolving from conventional assessment of RV dilatation and regional wall-motion abnormalities toward integrated characterization of ventricular mechanics and myocardial substrate. [44,45] 3D echocardiography, RV and RA strain, CMR feature-tracking, LGE, and parametric mapping provide complementary information on structural remodeling, myocardial deformation, fibrosis, and disease activity, with potential implications for both early diagnosis and arrhythmic risk stratification [44,47,49].

## 7. Congenital Heart Defects (Atrial Septum Defect)

Congenital intracardiac shunts produce a spectrum of right-heart remodeling determined by shunt location, magnitude, duration, and the development of pulmonary vascular disease. Among these lesions, atrial septal defect (ASD) represents the most extensively studied model of chronic RA and RV volume overload. Echocardiography remains the first-line imaging modality, whereas CMR provides reference-standard quantification of RV volumes and RVEF and enables accurate measurement of pulmonary-to-systemic flow ratio (Qp/Qs) [53].

3D echocardiography provides volumetric evaluation of RV remodeling and allows detailed visualization of the septal defect and its relationship with adjacent cardiac structures [54]. By reconstructing the entire RV chamber, 3D echocardiography enables assessment of RV end-diastolic and end-systolic volumes and RVEF while reducing the limitations imposed by the complex RV geometry [55]. Speckle-tracking echocardiography provides complementary assessment of RV myocardial deformation, although strain values in ASD are strongly influenced by loading conditions. Some cohorts have demonstrated augmented RV longitudinal deformation and hyperdynamic 3D RVEF during chronic volume overload, followed by a decrease after defect closure, whereas other studies have shown impaired RVGLS compared with healthy controls and subsequent improvement following unloading [55,56]. Importantly, persistent reduction in RV free-wall deformation has been reported decades after ASD repair, suggesting that subtle RV dysfunction may remain despite successful anatomical correction [54].

Advanced echocardiography may also identify RA remodeling: reduced RA longitudinal strain and abnormal 3D-derived atrial functional indices have been associated with subsequent paroxysmal atrial fibrillation [57].

CMR complements echocardiography by combining accurate RV volumetry with quantitative assessment of shunt physiology [58]. Conventional phase-contrast CMR enables calculation of Qp/Qs, while CMR feature-tracking provides quantitative evaluation of RV myocardial deformation [58,59]. In patients with PAH associated with ASD, impaired CMR-derived RVGLS has been detected even when RVEF remains preserved and correlates with RV volumes, filling pressures, and biomarkers of myocardial stress [59]. Four-dimensional (4D) flow CMR adds a comprehensive hemodynamic dimension by simultaneously depicting intracardiac flow pathways and quantifying pulmonary, systemic, and shunt-related flow within a single volumetric dataset. More recently, 4D-flow-derived hemodynamic force analysis has demonstrated abnormal RV flow forces in ASD that substantially normalize after shunt closure, providing a novel perspective on reverse remodeling following correction of chronic volume overload [58].

CMR tissue characterization may reveal structural myocardial remodeling beyond changes in RV size and function. In adults with unrepaired secundum ASD, LGE is frequently observed at the RVIPs, with more extensive septal and RV free-wall involvement in patients with PH or Eisenmenger physiology. Native T1 and ECV are also increased with advancing pulmonary vascular disease and are associated with higher pulmonary vascular resistance and impaired RVEF, suggesting progressive diffuse interstitial remodeling in addition to focal abnormalities detected by LGE. Importantly, elevated native T1 and ECV may be present even before overt PH develops. However, RVIPs’ LGE is not specific for irreversible fibrosis and may partly reflect mechanical stress related to chronic RV loading; similarly, native T1 and ECV are sensitive to extracellular expansion but are not specific for fibrosis alone [60].

The role of myocardial T2 mapping in uncomplicated intracardiac shunts remains insufficiently established. An emerging alternative approach exploits the sensitivity of blood T2 to oxygenation: the RV/LV blood-pool T2 ratio has been shown to be increased in left-to-right shunts and to correlate strongly with Qp/Qs, suggesting a potential rapid screening marker for occult shunting in patients presenting with otherwise unexplained RV dilatation. This technique should, however, be distinguished from myocardial T2 mapping and currently remains investigational [61].

Overall, advanced imaging of congenital intracardiac shunts is evolving from anatomical identification of the defect toward integrated characterization of right-heart remodeling and intracardiac hemodynamics. 3D echocardiography, RV and RA strain, CMR feature-tracking, phase-contrast and 4D-flow imaging, together with LGE and parametric mapping, provide complementary information on chamber adaptation, myocardial deformation, flow burden, and tissue remodeling and may improve assessment of disease progression and reverse remodeling after shunt closure [55,58,60].

## 8. Tricuspid Valve Disease

Tricuspid regurgitation (TR) has been re-appraised from a largely benign bystander lesion to an independent contributor to morbidity, right heart failure, and mortality [62]. With an estimated prevalence of clinically significant TR exceeding 1.6 million individuals in the United States alone [63], and the emergence of transcatheter tricuspid valve interventions (TTVIs)—including edge-to-edge repair devices (TriClip, PASCAL) and transcatheter tricuspid valve replacement (TTVR)—advanced cardiovascular imaging has become central to patient selection, procedural planning, intraprocedural guidance, and post-procedural assessment of RV reverse remodeling [62]. The RV’s complex geometry and load sensitivity make accurate imaging-based characterization of both valvular and ventricular function indispensable across all stages of clinical management [2,62].

TTE remains the cornerstone of TR assessment, with current guidelines recommending a multiparametric approach to severity quantification. Key parameters include vena contracta width, effective regurgitant orifice area (EROA) by the proximal isovelocity surface area (PISA) method, regurgitant volume, and the recently introduced ‘torrential’ category defined by EROA ≥ 0.8 cm^2^, which identifies patients at particularly high risk of adverse outcomes. Three-dimensional echocardiography is a valuable adjunct in patients considered for TTVI, providing detailed assessment of tricuspid annular dimensions and geometry, leaflet morphology, and the coaptation gap, particularly for procedural planning. TEE with real-time 3D reconstruction provides the reference standard for intraoperative and intraprocedural guidance. RV function—assessed by 3D RVEF, TAPSE, RVFWLS, and the TAPSE/sPAP ratio as a surrogate of RV–PA coupling—represents a key determinant of procedural eligibility and expected clinical benefit. RA dilatation further reflects chronic hemodynamic burden and is associated with reduced likelihood of RV reverse remodeling after intervention [2,62].

CMR provides accurate and reproducible assessment of RV volumes and function and can complement echocardiography in the quantitative evaluation of TR. Conventional CMR quantification is commonly performed indirectly by subtracting pulmonary arterial forward flow, measured using phase-contrast imaging, from RV stroke volume, allowing calculation of tricuspid regurgitant volume and fraction. Direct quantification across the moving tricuspid valve remains technically more challenging, although advanced 4D-flow CMR may facilitate direct valve-plane flow assessment [64].

Observational studies have identified CMR-derived indices of adverse RV remodeling, including reduced RVEF ≤ 45% and increased RVEDVI > 110 mL/m^2^ as markers associated with worse clinical outcomes. This cut-off value of RVEF has been used to define global RV dysfunction in selected TTVI cohorts. Thus, both should not be interpreted as universally validated guideline thresholds [65]. Serial CMR assessment is the reference modality for evaluating RV reverse remodeling following TTVI, with post-procedural improvement in RVEF and reduction in RVEDVI constituting the principal evidence of hemodynamic benefit [66]. Tissue characterization with T1 mapping, ECV fraction, and LGE may identify diffuse RV interstitial fibrosis or focal myocardial injury that limits the extent of post-interventional functional recovery and may have prognostic implications in patients with advanced RV remodeling at baseline [65].

## 9. Right-Sided Cardiac Tumors

Cardiac masses involving the right heart represent a heterogeneous group of benign and malignant neoplasms, metastatic lesions, and tumor-like structures. Although most primary cardiac tumors are benign, the right-sided location of a cardiac mass should raise particular concern for malignancy, as malignant primary cardiac tumors show a greater predilection for the right heart than benign lesions. Secondary cardiac tumors are considerably more common than primary malignant cardiac neoplasms and may reach the right heart by hematogenous spread, direct extension, or intravascular propagation through the venae cavae. Therefore, identification of a right-sided cardiac mass should prompt evaluation for both primary and extracardiac malignancy. Echocardiography remains the first-line imaging modality, providing real-time assessment of mass location, size, mobility, attachment, hemodynamic consequences, and associated pericardial effusion. TEE is particularly useful for small, highly mobile, or valvular lesions, whereas RT3D echocardiography may improve visualization of tumor morphology, attachment, and spatial relationships. Contrast-enhanced echocardiography provides additional information on mass vascularity and may help differentiate avascular thrombi from vascularized tumors. CMR provides complementary information by enabling detailed tissue characterization and assessment of local invasion [67]. Multiparametric CMR, incorporating T1- and T2-weighted imaging, first-pass perfusion, and LGE, can help distinguish thrombus from neoplastic tissue and may provide additional clues regarding the benign or malignant nature of a cardiac mass [68].

Among benign right-heart tumors, cardiac myxoma and papillary fibroelastoma may benefit from advanced multimodality imaging. 3D echocardiography improves visualization of tumor morphology, attachment, stalk, and spatial relationships with adjacent cardiac structures. Cardiac myxoma may occasionally arise in the RA, although LA localization is substantially more common. Papillary fibroelastomas may involve the tricuspid or, more rarely, pulmonary valve and are usually small, highly mobile valvular lesions for which echocardiography, particularly TEE, has an advantage because of its high temporal and spatial resolution [67]. In cardiac myxoma, CMR provides additional tissue characterization. Parametric studies have demonstrated increased native T1, T2, and ECV, whereas LGE is typically heterogeneous, consistent with the variable water content, extracellular matrix expansion, and heterogeneous histological composition of these tumors. Myxomas typically show heterogeneous LGE, consistent with their variable myxoid, fibrotic, hemorrhagic, and necrotic composition [69]. CMR characterization of papillary fibroelastoma is more challenging because of its small size and high mobility; however, individual reports have demonstrated prolonged T1 and T2 values and marked delayed enhancement [70]. Thus, while 3D echocardiography is particularly valuable for anatomical characterization of these benign tumors, multiparametric CMR may provide complementary information regarding tissue composition, although evidence for quantitative mapping in papillary fibroelastoma remains limited [67,69,70].

Angiosarcoma is the most characteristic primary malignant tumor of the right heart and typically originates from the RA free wall as a broad-based, heterogeneous, and infiltrative mass. 3D echocardiography improves delineation of the tumor base, spatial relationships, and invasion of adjacent structures, and can also facilitate biopsy or surgical planning. Contrast-enhanced echocardiography may demonstrate tumor vascularity, which can help distinguish angiosarcoma from avascular thrombus. Speckle-tracking-derived RV or RA strain may provide complementary information on the functional consequences of tumor infiltration or pressure/volume loading; however, its diagnostic role in angiosarcoma remains insufficiently established and no characteristic strain pattern has been validated. CMR is particularly valuable for defining tumor extent and tissue composition. On CMR, the heterogeneous composition of cardiac angiosarcoma results in variable T1- and T2-weighted signal characteristics, with intratumoral hemorrhage and necrosis contributing to marked tissue heterogeneity. Its rich vascular supply is typically reflected by prominent first-pass enhancement, while delayed imaging frequently demonstrates an irregular LGE pattern with enhancing viable or fibrotic components interspersed with non-enhancing necrotic areas [67,71].

Primary cardiac lymphoma (PCL) is an important malignant cardiac neoplasm with a marked predilection for the RA and RV [72,73]. 3D echocardiography may provide additional information by improving spatial visualization of tumor infiltration and its relationship with adjacent right-heart structures, while also enabling quantitative assessment of tumor volume and burden [67]. Recent observations further suggest that serial 3D echocardiography may characterize tumor-related alterations in tricuspid annular geometry [74]. Contrast-enhanced echocardiography may complement morphological assessment by evaluating tumor perfusion and facilitating differentiation from avascular thrombus. Although speckle-tracking assessment of RA and RV function may theoretically quantify the functional consequences of myocardial infiltration, no lymphoma-specific RV or RA strain pattern has yet been established [67]. CMR complements echocardiography by providing multiparametric tissue characterization and detailed assessment of myocardial and pericardial infiltration. PCL most commonly appears as an infiltrative right-sided mass with intermediate or isointense signal on T1-weighted imaging and mildly increased to high signal on T2-weighted sequences, although considerable heterogeneity has been reported. First-pass perfusion and LGE are variable, with enhancement frequently observed, while diffusion-weighted imaging may demonstrate restricted diffusion related to the high cellularity of lymphoma [72]. Emerging parametric CMR techniques may provide further tissue characterization; isolated reports have demonstrated increased native T1 and T2 values in active cardiac lymphoma, although standardized T1- or T2-mapping profiles for PCL have not yet been established. Therefore, the major contemporary role of CMR lies in combining morphology, cine imaging, T1/T2 tissue characteristics, perfusion, diffusion, and LGE to characterize tumor composition and extent rather than relying on a single imaging biomarker [72,74].

A major diagnostic challenge is the differentiation of right-heart tumors from thrombi and pseudomasses. Advanced echocardiographic techniques may improve morphological and functional characterization of intracardiac masses. 3D echocardiography provides more accurate visualization of mass morphology, attachment, mobility, volume, and spatial relationships with adjacent right-heart structures, which may facilitate differentiation between sessile or infiltrative tumors and intracavitary thrombi. Contrast-enhanced echocardiography adds information on vascularity and perfusion: thrombi are typically avascular and show absent or minimal contrast enhancement, whereas neoplastic masses, particularly malignant tumors, usually demonstrate partial or marked enhancement depending on their vascularity. Speckle-tracking echocardiography may additionally quantify secondary impairment of RA or RV mechanics caused by tumor infiltration or hemodynamic obstruction, although it currently has no established role in distinguishing tumors from thrombi [67]. CMR provides a major advantage through tissue characterization: thrombi generally demonstrate absent or minimal first-pass perfusion and lack LGE, whereas most tumors show varying degrees of vascularity and contrast enhancement. CMR therefore provides high diagnostic accuracy for differentiating thrombus from tumors, while long-inversion-time delayed-enhancement imaging may be particularly useful for identifying RA thrombi [68].

## 10. RV Involvement in Cardiac Amyloidosis

Cardiac amyloidosis (CA), and in particular transthyretin amyloid cardiomyopathy (ATTR-CA) in both its wild-type (ATTRwt) and hereditary (ATTRv) forms, represents a significantly underdiagnosed infiltrative cardiomyopathy with important right heart involvement. ATTR-CA is now recognized as a common and frequently overlooked etiology of HFpEF in the elderly population, and right heart compromise is a critical determinant of prognosis. The development of disease-modifying pharmacotherapies—including tafamidis, RNA-interference agents (patisiran, vutrisiran), and investigational CRISPR-based TTR gene silencing strategies—has fundamentally changed the clinical trajectory of ATTR-CA and created an urgent need for sensitive, reproducible imaging biomarkers capable of early disease detection, staging, risk stratification, and pharmacodynamic monitoring of therapeutic response [75].

The classical echocardiographic appearance of CA encompasses concentric biventricular hypertrophy, a granular sparkling myocardial texture, biatrial enlargement, and impaired diastolic function with restrictive filling physiology. The apical sparing longitudinal strain pattern—characterized by relative preservation of apical contractility with basal and mid-ventricular impairment, producing a bull’s eye plot appearance colloquially described as ‘cherry on top’—is a sensitive and specific echocardiographic signature of CA that distinguishes it from hypertensive heart disease or hypertrophic cardiomyopathy [75]. RV involvement in CA is common and prognostically significant: RV free wall thickening, elevated RV systolic pressure, reduced RVGLS, and reduced TAPSE independently predict mortality and heart-failure hospitalization in ATTR-CA. Reduced tricuspid annular tissue Doppler velocities (TDI S’) further reflect RV myocardial infiltration [2,75]. The TAPSE/sPAP ratio, as a non-invasive measure of RV–PA uncoupling, may identify patients with disproportionate hemodynamic compromise relative to their degree of amyloid burden and carries independent prognostic value [2].

CMR provides comprehensive non-invasive tissue characterization of CA. LGE in CA follows a characteristic pattern of diffuse subendocardial or transmural circumferential enhancement with difficulty achieving myocardial nulling on standard inversion recovery sequences, reflecting global extracellular gadolinium retention due to amyloid fibril infiltration. T1 mapping and extracellular volume (ECV) fraction quantification represent the most sensitive CMR measures of amyloid burden, demonstrating strong correlations with endomyocardial biopsy-derived amyloid load, circulating NT-proBNP, and adverse clinical outcomes. Importantly, CMR tissue characterization extends to the RV free wall: elevated native T1 and expanded ECV in the RV myocardium can detect early infiltration before the development of overt RV wall thickening or systolic dysfunction, providing a window for pre-emptive therapeutic escalation. Serial ECV quantification is emerging as a pharmacodynamic biomarker in the context of tafamidis therapy and RNA-interference agents, with preliminary data suggesting that treatment-related reductions in ECV parallel clinical and biomarker improvement, potentially establishing CMR as a tool for non-invasive monitoring of disease modification at the tissue level [75].

## 11. Future Directions and Emerging Technologies

Artificial intelligence (AI) and machine learning are poised to transform the clinical workflow in right heart imaging. Deep learning algorithms for automated RV segmentation from both echocardiographic and CMR datasets have demonstrated accuracy comparable to expert human readers, with substantially reduced analysis time. Beyond segmentation, AI-based pipelines that integrate multi-parametric imaging features—including RV strain, volumetric indices, and tissue characterization—with clinical, biomarker, and hemodynamic variables hold promise for individualized risk stratification in PAH and ARVC. Federated learning frameworks, enabling model training across institutions without sharing patient data, may further accelerate the development and external validation of such tools.

Four-dimensional (4D) flow CMR, which acquires time-resolved three-dimensional velocity fields across the entire cardiovascular volume, offers unique opportunities to characterize complex flow patterns in the right heart and pulmonary vasculature. Metrics such as pulmonary arterial wall shear stress, vorticity, and kinetic energy loss are emerging as sensitive hemodynamic biomarkers in PH that are not accessible by conventional modalities [76,77,78,79]. Complementarily, exercise CMR—performed on dedicated MR-compatible ergometers—enables direct assessment of RV contractile reserve and RV–PA coupling under physiological stress. This approach is particularly valuable in unmasking early hemodynamic impairment in patients with exertional dyspnea but resting parameters within normal limits, including exercise-induced PH and early-stage HFpEF [35].

Although echocardiography and CMR constitute the primary focus of this review, several emerging complementary modalities may further extend right-heart phenotyping and are briefly discussed here to provide perspective on future multimodality imaging strategies. Hybrid positron emission tomography–CMR (PET-CMR) integrates functional metabolic information with high-resolution structural and tissue characterization in a single examination. In ARVC and cardiac sarcoidosis, simultaneous assessment of myocardial inflammation and fibrosis by PET-CMR may refine diagnostic accuracy and guide immunosuppressive therapy decisions. In PAH, hybrid PET/MRI has demonstrated independent prognostic utility: Kazimierczyk et al. showed that myocardial [18F] FDG uptake reflects RV metabolic adaptation to pressure overload and independently predicts adverse clinical outcomes. Follow-up studies further demonstrated that targeted PAH-specific therapy modulates cardiac fluorodeoxyglucose distribution on serial imaging, supporting PET/MRI as a functional biomarker of therapeutic response. Additionally, the RV cardiac power index was found to be independently associated with myocardial glucose metabolism and prognosis in PAH, underscoring the prognostic value of integrated metabolic–structural imaging in risk stratification of this patient population [76,77,78]. Photon-counting detector computed tomography (PCD-CT), a next-generation CT technology offering improved spatial resolution, reduced noise, and intrinsic spectral capability, promises enhanced characterization of cardiac masses—including differentiation of lipomatous from fibrous components—and superior delineation of thromboembolic burden and vascular remodeling in CTEPH, potentially reducing the need for invasive catheter-based procedures [80,81].

Reconstructed CMR-based pressure–volume loop analysis may provide estimates of RV contractile and coupling parameters, including end-systolic elastance and the Ees/Ea ratio (or its inverse, Ea/Ees). However, such non-invasive or single-beat reconstructions remain model-dependent and should not be considered equivalent to direct multi-beat invasive pressure–volume assessment. These metrics offer a more mechanistically complete characterization of RV-PA coupling than isolated volume or EF parameters and may improve prognostication and the monitoring of therapeutic responses in PH [35,36]. Additionally, RA strain derived from feature-tracking CMR, and 2D speckle-tracking echocardiography is gaining recognition as a sensitive marker of RA remodeling and elevated filling pressure across a spectrum of right heart diseases [2]. RA reservoir strain correlates with pulmonary capillary wedge pressure and RV diastolic dysfunction, offering incremental diagnostic and prognostic value beyond conventional chamber-size measurements [40]. AI applied to both echocardiography and CMR can facilitate automated RV contouring and quantification of chamber volumes, RVEF, and, increasingly, myocardial deformation, substantially reducing analysis time and potentially decreasing inter-observer variability. This may be particularly relevant for the RV, whose complex geometry and prominent trabeculation make manual delineation challenging. These techniques should currently be considered complementary to expert interpretation, while multicenter validation and methodological harmonization remain essential before their broader incorporation into routine right-heart assessment [82].

## 12. Conclusions

Advanced cardiovascular imaging with echocardiography and CMR has fundamentally transformed the evaluation of the right heart and pulmonary circulation. Across all the diseases and pathologies reviewed, both modalities provide complementary, clinically actionable information on right heart morphology, function, myocardial tissue characteristics, and hemodynamics (Table 1). Echocardiography remains the first-line modality, offering real-time assessment with high temporal resolution, while CMR provides superior anatomical delineation, tissue characterization through LGE and parametric mapping, and accurate volumetric and flow quantification. The integration of novel techniques—including RV and RA strain, three-dimensional volumetrics, 4D flow CMR, and machine learning-assisted analysis—is expanding the diagnostic and prognostic precision of right heart imaging. A multimodality imaging approach, tailored to the clinical context and individual patient characteristics, represents the current standard of care and the foundation for future advances in the field.

## Figures and Tables

**Table 1:** Consolidated summary of echocardiographic and CMR imaging characteristics across the nine cardiopulmonary disease entities discussed in this review. Abbreviations are defined in the List of Abbreviations.

Disease	Key Echocardiographic Parameters	Key CMR Parameters	Novel/Emerging Techniques	Clinical Role/Key Finding
Pulmonary Arterial Hypertension (PAH)	RV enlargement; RV/LV ratio; septal flattening; RA area; IVC dilatation; FAC; TAPSE; S′; TRV; RVOT AT; pericardial effusion;	RVEF (reference standard for RV volumes); RV volumes; LGE at RVIPs; T1 and T2 mapping; ECV fraction; RV-PA coupling indices	TAPSE/sPAP ratio; RV free-wall longitudinal strain (RVFWLS); 3D RVEF; RVGLS; PET/MRI (FDG myocardial metabolic imaging; independent prognostic value)	Diagnosis; risk stratification; treatment monitoring; prognosis
Pulmonary Embolism (PE)	RV dilatation; RV/LV ratio; McConnell sign; 60/60 sign (PA AT < 60 ms + sPAP < 60 mmHg); TAPSE; FAC	RVEF and RV volumes (reference standard); phase-contrast pulmonary flow; limited role in acute setting	RVGLS; RVFWLS; 3D RVEF; RA reservoir/conduit strain; T1/ECV for post-PE myocardial remodeling assessment	Acute risk stratification; RV dysfunction severity; treatment response; post-PE monitoring
Chronic Thromboembolic PH (CTEPH)	TRV; RV dilatation; septal flattening; TAPSE; RVOT AT with mid-systolic notching; exercise stress echocardiography	RVEF; RVEDVI; phase-contrast flow (PA compliance, net pulmonary flow); LGE at RVIPs; T1/ECV mapping	RVGLS (pre/post-PEA prognostic marker); T1/ECV (reverse remodeling monitoring after BPA); exercise CMR	Pre-interventional risk stratification; post-PEA/BPA RV reverse remodeling monitoring
Heart Failure with Preserved EF (HFpEF)	TAPSE; FAC; RVFWLS; TAPSE/sPAP ratio (RV-PA coupling surrogate); exercise echocardiography (RV contractile reserve); 3D RVEF/sPAP ratio	RVEF (prognostic); RVGLS and RVGCS (CMR feature-tracking); RA reservoir/conduit strain; RV ECV (T1 mapping)	RV ECV fraction; exercise CMR (stress RV-PA coupling); 3D RVEF/sPAP; RA feature-tracking strain	RV phenotyping in HFpEF; prognosis; identification of PH-HFpEF subgroup
Arrhythmogenic Right Ventricular Cardiomyopathy (ARVC)	3D RVEF; 3D RV volumes; RVGLS; regional deformation (basal/mid RVFW); mechanical dispersion; RA reservoir/conduit/booster strain	LGE (focal fibrosis—major Task Force criterion); RVEF; RV volumes; regional wall motion; T1 mapping; ECV	CMR feature-tracking RVGLS (diagnostic and prognostic); ECV (diffuse fibrosis, arrhythmic risk); T2 mapping (inflammatory ‘hot phase’)	Diagnosis (Task Force criteria); arrhythmic risk stratification; SCD prevention
Congenital Heart Defects with Intracardiac Shunts (ASD)	RV enlargement; 3D RVEF; RVGLS; RA strain; septal anatomy and defect characterization	Qp/Qs (phase-contrast CMR); RV volumetry; feature-tracking RVGLS; 4D flow CMR; LGE at RVIPs; T1/ECV mapping	4D flow hemodynamic force analysis; T2 blood pool RV/LV ratio (occult shunt screening); CMR feature-tracking	Shunt quantification; RV volume overload assessment; post-closure reverse remodeling evaluation
Tricuspid Valve Disease	Vena contracta; EROA (PISA); regurgitant volume; 3D annular dimensions and geometry (TTVI planning); TAPSE; RVFWLS; TAPSE/sPAP ratio; RA dilatation	Regurgitant fraction (phase-contrast); RVEF; RVEDVI; T1 mapping; ECV; LGE	Serial CMR for post-TTVI RV reverse remodeling; tissue characterization (T1/ECV/LGE) for pre-interventional risk	TR severity quantification; TTVI candidate selection; post-intervention RV monitoring
Right-Sided Cardiac Tumors	Mass size, mobility, and attachment; 3D morphology; contrast-enhanced echo (vascularity); TEE; RA/RV strain (functional consequences)	T1/T2-weighted tissue signal; first-pass perfusion; LGE pattern; DWI (lymphoma); multiparametric tissue characterization	Multiparametric CMR mapping (T1/T2 + LGE + perfusion + DWI); photon-counting CT (PCD-CT) for mass characterization	Tumor vs. thrombus differentiation; tissue characterization; local invasion and malignancy staging
RV Involvement in Cardiac Amyloidosis	Biventricular hypertrophy; granular myocardial texture; biatrial enlargement; apical sparing GLS pattern (‘cherry on top’); RVGLS; TAPSE; TDI S′; TAPSE/sPAP ratio	Diffuse subendocardial/circumferential LGE (difficulty nulling); T1 mapping; ECV fraction (amyloid burden and treatment monitoring); RV free wall T1/ECV (early infiltration detection)	Serial ECV for pharmacodynamic monitoring of tafamidis/RNA-interference agents; RV ECV for pre-clinical infiltration detection	Diagnosis; staging; risk stratification; therapeutic monitoring of disease-modifying therapy

## Data Availability

No new data were created or analyzed in this study. Data sharing is not applicable to this article.

## Abbreviations

The following abbreviations are used in this manuscript:

2D	two-dimensional
3D	three-dimensional
4D	four-dimensional
AI	artificial intelligence
ARVC	arrhythmogenic right ventricular cardiomyopathy
ASD	atrial septal defect
ATTR-CA	transthyretin amyloid cardiomyopathy
ATTRv	variant (hereditary) transthyretin amyloidosis
ATTRwt	wild-type transthyretin amyloidosis
BPA	balloon pulmonary angioplasty
CA	cardiac amyloidosis
CMR	cardiovascular magnetic resonance
CRISPR	clustered regularly interspaced short palindromic repeats
CT	computed tomography
CTEPH	chronic thromboembolic pulmonary hypertension
CTPA	computed tomography pulmonary angiography
Ea/Ees	arterial elastance/end-systolic ventricular elastance ratio
ECV	extracellular volume fraction
EF	ejection fraction
EROA	effective regurgitant orifice area
FAC	fractional area change
[18F] FDG	fluorine-18 fluorodeoxyglucose
GLS	global longitudinal strain
HFpEF	heart failure with preserved ejection fraction
IVC	inferior vena cava
LA	left atrium
LGE	late gadolinium enhancement
LV	left ventricle
LVEF	left ventricular ejection fraction
MR	magnetic resonance
MRI	magnetic resonance imaging
NT-proBNP	N-terminal pro-B-type natriuretic peptide
PAH	pulmonary arterial hypertension
PARAGON-HF	Prospective Comparison of Angiotensin Receptor–Neprilysin Inhibitor with Angiotensin Receptor Blocker Global Outcomes in Heart Failure with Preserved Ejection Fraction
PCL	primary cardiac lymphoma
PCD-CT	photon-counting detector computed tomography
PE	pulmonary embolism
PEA	pulmonary endarterectomy
PET-CMR	positron emission tomography–cardiovascular magnetic resonance
PET/MRI	positron emission tomography/magnetic resonance imaging
PH	pulmonary hypertension
PH-HFpEF	pulmonary hypertension associated with heart failure with preserved ejection fraction
PISA	proximal isovelocity surface area
P-V	pressure-volume
Qp/Qs	pulmonary-to-systemic blood flow ratio
RA	right atrium
RAGLS	right atrial global longitudinal strain
RA–RV	right atrial–right ventricular
RNA	ribonucleic acid
RT3D	real-time three-dimensional echocardiography
RT3DE	real-time three-dimensional echocardiography
RV	right ventricle/right ventricular
RV/LV	right ventricular-to-left ventricular ratio
RV–PA	right ventricular–pulmonary arterial
RVEDVI	right ventricular end-diastolic volume index
RVEF	right ventricular ejection fraction
RVEF/sPAP	right ventricular ejection fraction-to-systolic pulmonary artery pressure ratio
RVFWLS	right ventricular free-wall longitudinal strain
RVGCS	right ventricular global circumferential strain
RVGLS	right ventricular global longitudinal strain
RVGLS/sPAP	right ventricular global longitudinal strain-to-systolic pulmonary artery pressure ratio
RVIPs	right ventricular insertion points
RVOT AT	right ventricular outflow tract acceleration time
RV S′	peak systolic velocity of the tricuspid annulus
sPAP	systolic pulmonary artery pressure
T1	longitudinal relaxation time
T2	transverse relaxation time
TAPSE	tricuspid annular plane systolic excursion
TAPSE/sPAP	tricuspid annular plane systolic excursion-to-systolic pulmonary artery pressure ratio
TDI	tissue Doppler imaging
TEE	transesophageal echocardiography
TR	tricuspid regurgitation
TRJV	tricuspid regurgitant jet velocity
TRV	tricuspid regurgitation velocity
TTE	transthoracic echocardiography
TTR	transthyretin
TTVI	transcatheter tricuspid valve intervention
TTVR	transcatheter tricuspid valve replacement
WHO	World Health Organization
